# Substrate-Specific Inactivation of Human AAG by Tumor-Associated Single Nucleotide Polymorphic Variants

**DOI:** 10.3390/ijms27156595

**Published:** 2026-07-24

**Authors:** Olga A. Kladova, Timofey E. Tyugashev, Artemiy S. Bakman, Aleksandra A. Kuznetsova

**Affiliations:** Knorre Institute of Chemical Biology and Fundamental Medicine, Siberian Branch of Russian Academy of Sciences, Novosibirsk 630090, Russia; tyugashev@1bio.ru (T.E.T.); art-bakman@yandex.ru (A.S.B.)

**Keywords:** alkyladenine DNA glycosylase (AAG, MPG), base excision repair, single nucleotide polymorphism (SNP), substrate specificity, cancer biomarker, molecular dynamics

## Abstract

Human alkyladenine DNA glycosylase (AAG) initiates base excision repair of various alkylated and deaminated purines. Single nucleotide polymorphisms (SNPs) in the AAG gene occur frequently in populations and tumors, but the functional impact of most variants remains unknown. Previously, we identified three SNPs—P94L, V158M, and E293K—which have been predicted to have a high deleterious potential. This study aimed to characterize their biochemical properties and structural consequences. Using a combination of biochemical assays and molecular dynamics simulations, we assessed the thermal stability, DNA binding affinity, and catalytic activity of these mutants on two structurally distinct substrates: 1, N^6^-ethenoadenosine (εA) and hypoxanthine (Hx). All three mutants exhibited reduced melting temperatures, indicating pronounced destabilization. Despite this, their DNA-binding affinities remained close to WT AAG. Strikingly, the mutants displayed differential loss of catalytic activity: P94L was inactive against both εA and Hx; V158M retained activity against εA but lost activity against Hx; and E293K was active against Hx but inactive against εA. MD simulations revealed that P94L alters the flexible R138–T143 loop, V158M narrows the active site cleft, and E293K disrupts a C-terminal salt bridge while increasing DNA engagement by the positively charged tail. These findings demonstrate that non-active-site SNPs can qualitatively reprogram the substrate specificity of AAG. These variants could be considered as potential functional biomarkers for cancer risk and response to alkylating chemotherapy.

## 1. Introduction

Maintaining genome integrity is one of the most important functions of the cell. Its disruption can lead to accumulation of mutations and genomic instability, which may contribute to the development of cancer and neurodegenerative diseases [1]. DNA damage occurs constantly under the influence of both endogenous metabolites, such as reactive oxygen species, and replication errors, as well as environmental factors [2,3,4,5,6]. Among the various types of damage, alkylated bases have a special significance. If not corrected, these modifications can lead to mutations during replication, resulting in base pair substitutions and, consequently, malignant transformation [7,8,9].

A key component in the cellular defense against alkylating stress is the enzyme alkyladenine DNA glycosylase (AAG, also known as MPG and N-methylpurine DNA glycosylase) [10,11,12,13]. AAG is part of the base excision repair pathway, which initiates the repair process by excising a wide range of alkylated DNA base derivatives, including 3-methyladenine, 7-methyladenine, 3-methylguanine, hypoxanthine and etheno adducts [14,15,16,17]. After removing the damaged base, AAG leaves an apurinic/apyrimidinic site that is then processed by subsequent repair enzymes [12,18].

Despite its protective role, the activity of AAG must be carefully balanced. Both deficiency and overexpression of this enzyme can have negative consequences [19,20,21,22,23]. Elevated levels of AAG have been observed in various malignancies, including glioma [24], breast [25] and lung cancer [19,20]. Furthermore, excessive glycosylase activity can also lead to the formation of toxic DNA repair intermediates (single strand DNA breaks), making AAG a potential target for therapeutic inhibition [26,27,28].

As whole-genome sequencing data accumulates, it has become clear that single nucleotide polymorphisms (SNPs) in the *AAG* gene occur at a significant frequency in the population. However, the functional significance of most of these substitutions remains uncharacterized [29,30,31]. Furthermore, tumor-associated AAG variants have been identified in cancer databases (e.g., COSMIC, National Cancer Institute database, cBioPortal), but it remains unclear whether these substitutions represent driver events or passenger mutations with a neutral effect. There is evidence that even non-conservative amino acid substitutions, located far from the active site of the enzyme, can modulate catalytic efficiency by altering substrate binding constant or maximum reaction rates [29,32].

**Figure 1:**
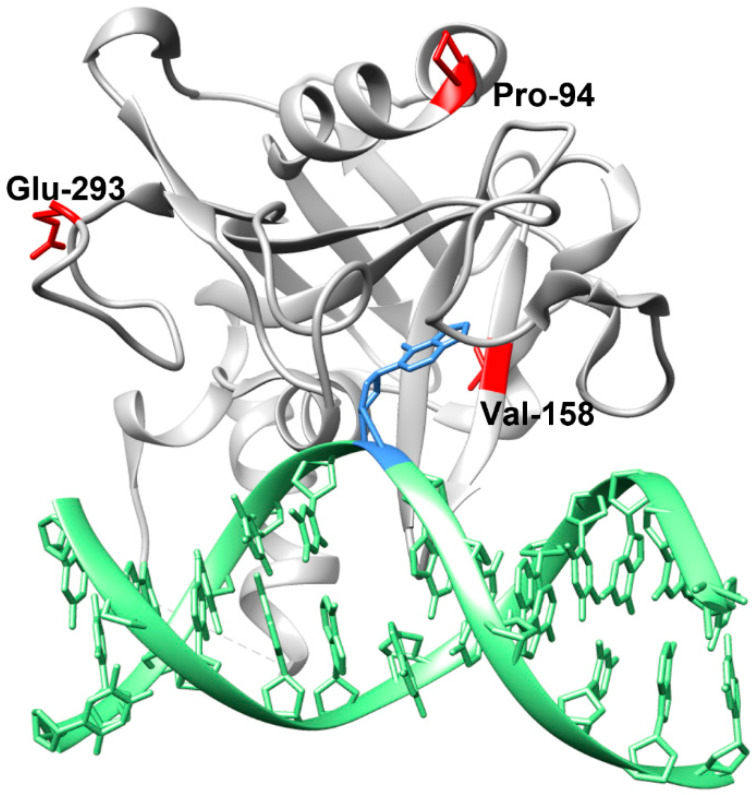
AAG complex with DNA containing 3, N^4^-ethenocystosine. The enzyme and substituted amino acid residues are colored in gray and red, respectively. DNA is shown in green, and the damaged base is shown in blue. PDB ID: 3QI5.

Previously, we analyzed known single nucleotide mutations in the *AAG* gene using bioinformatics approaches and ranked them according to their degree of negative impact on enzyme function [33]. In this study, we analyzed three rare polymorphisms (SNPs) of AAG that have a high probability of negative impact on function: P94L (rs772837868), V158M (rs562548652) and E293K (rs1377338969) (Figure 1).

The P94 residue, which is partially conserved across animal AAG orthologs, marks the beginning of the α2-helix. Notably, in bacterial, protozoan, or plant AAG homologues, the proline is not conserved, and the corresponding position is replaced by a polar amino acid residue [34,35,36,37].

The V158 residue is located in the middle of a conserved YXY motif, where the side chain of X (A, V, T, or L) is oriented outward, packed against the short α8-helix. The side chains of the flanking tyrosines are positioned in the active site of glycosylase. The Y157 side chain maintains a hydrogen bond with the H136 side chain, stabilizing the flipped-out base through a stacking interaction and a hydrogen bond with its phosphate group. The Y159 side chain is engaged in an edge-to-face interaction with the flipped-out base and forms a direct hydrogen bond with a phosphate oxygen atom [38].

The E293 residue belongs to the α10-helix in the C-terminal region of AAG, relatively poorly ordered. In most available crystal structures, this segment adopts an α-helical conformation. However, the last few C-terminal residues are not well-defined [39,40]. In the lower-affinity complex, electron density is missing for all residues after D289 [39]. The side chain of E293 can form hydrogen bond interactions with the side chain of R290, the amide group of D289, the side chain of Y278, and, through a persistent water bridge, the amide backbone of G281. Additionally, its backbone carbonyl group interacts with the side chain of R280. These interactions seem to stabilize the α10-helix within the protein globule. A previous systematic deletion analysis in murine AAG established that a variant retaining C-terminal residues up to Q315 (equivalent to Q294 in human AAG) retained enzymatic activity similar to that of the full-length protein. However, complete deletion of the α10-helix resulted in decreased solubility and loss of activity [41]. On the other hand, the E293 residue and the entire α10-helix have no direct counterparts in previously investigated distant orthologues [35,36,37].

A prior MMS sensitivity assay screening identified several substitutions, including P94A, P94L, P94Q, V158A, V158L, V158M, E293G and E293V as tolerated [42]. However, according to oncology databases, polymorphic variants such as P94L, V158M and E293K have been found in different types of tumors including endometrioid carcinoma, kidney carcinoma and cervix carcinoma (https://cancer.sanger.ac.uk/cosmic (accessed on 10 February 2025), https://www.cbioportal.org (accessed on 10 February 2025), https://portal.gdc.cancer.gov (accessed on 10 February 2025)). These findings suggest that these amino acid residues may be important for maintaining the function of the enzyme. The non-obvious functional position of these residues combined with their high probability of a negative impact on the enzyme function and their occurrence in cancer databases make them suitable candidates for further analysis.

## 2. Results and Discussion

### 2.1. Thermal Stability

**Figure 2:**
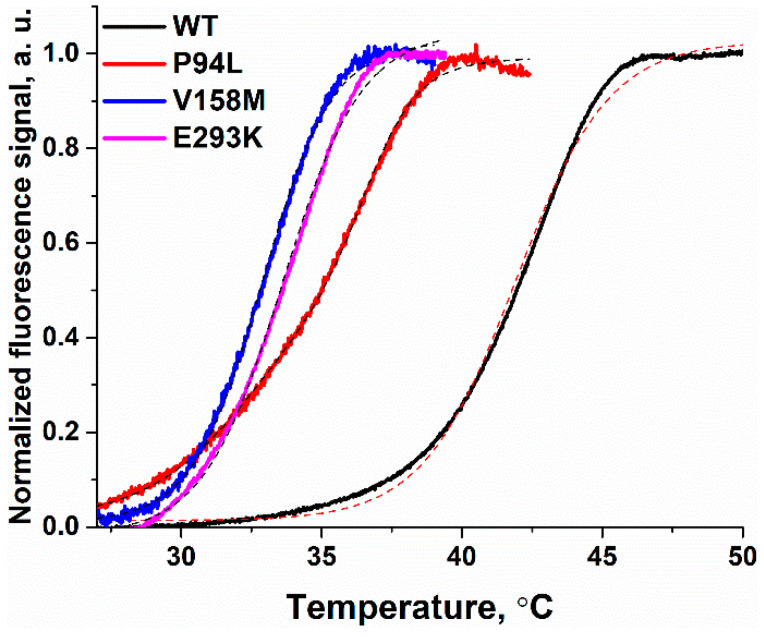
Thermal melting profiles for WT AAG and its mutants. WT AAG is shown in black, P94L in red, V158M in blue, and E293K in magenta. The calculated fitted lines are indicated as dashed lanes.

To evaluate the effect of the mutations on the enzyme’s structure, their melting temperatures were measured (Figure 2). The melting point of the WT enzyme was 42 °C, while all three SNP variants exhibited significantly lower melting temperatures, with reductions of 7–10 °C (Table 1).

Unsurprisingly, all three mutations are destabilizing. The P94L substitution replaces the N-capping proline with a bulky aliphatic amino acid, disrupting the P94–P97 stacking interaction. The V158M substitution disrupts hydrophobic contacts within the tightly packed conserved core, and the E293K eliminates a salt bridge. In contrast, stability predictions substantially underestimated the mutational effects. HotMuSiC [43] estimated ΔT_m_ decreases between 0.6 and 3.3 °C, while FoldX [44] and DynaMut [45] predicted ΔΔG shifts between 0.1 and 2.5 kcal/mol and only −0.65 to 0.65 kcal/mol, respectively.

**Figure 3:**
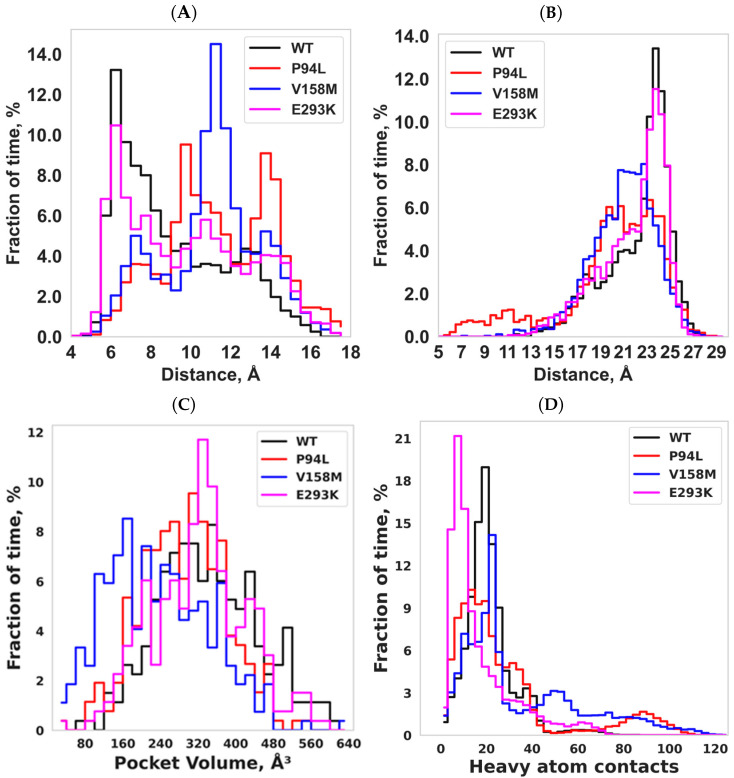
Distance between the Cα atoms of G140 and G176 (**A**), distance between the Cα atoms of G140 and F162 (**B**), active site cleft volume (**C**), and heavy atom contacts between the C-terminal region (aa 289–298) and the rest of the protein globule (aa 82–260) (**D**).

**Figure 4:**
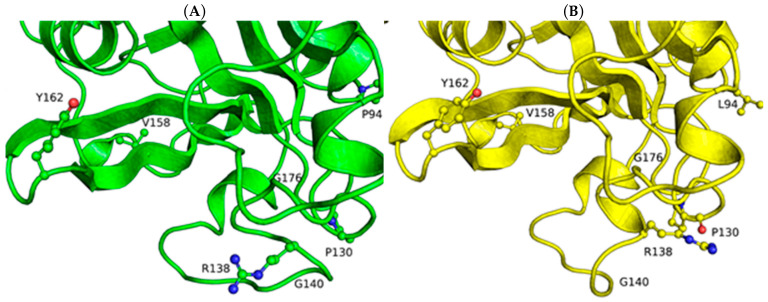

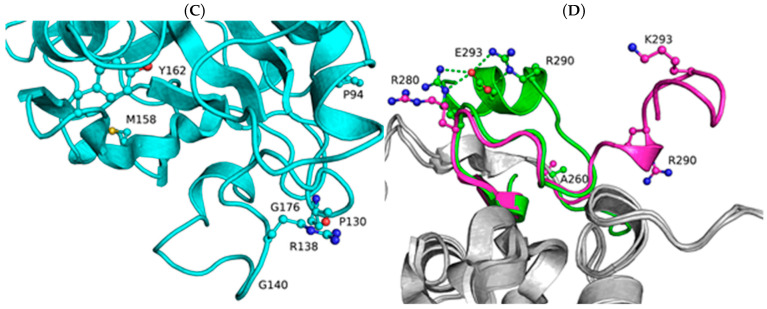
Apoenzyme AAG active site: (**A**) WT (green), (**B**) P94L (yellow), (**C**) V158M (cyan), and (**D**) an overlay of WT (green) and E293K (pink) C-terminal region snapshots.

In the apoenzyme simulations, the P94L substitution, despite introducing a bulky hydrophobic side chain in place of the more compact and rigid proline residue, results in only subtle, sub-angstrom shifts in the local environment of the enzyme. These effects are manifested further down the sequence, affecting the neighboring flexible loop, which engages both the lesion and the complementary DNA strand in the protein–DNA complex. The R138 side chain flips from a solvent-oriented position to maintain a stable hydrogen bond with the backbone oxygen atom of P130. The entire R138-T143 loop moves towards the apoenzyme’s active site cleft, as indicated by a decreased distance between the Cα atom of G140 and the Cα atom of the DNA-intercalating Y162 residue, and, in turn, an increased distance between the Cα atoms of G140 and G176 residues (Figure 3A,B and Figure 4A,B).

The V158M substitution introduces a steric clash between the larger M158 side chain and the α8-helix backbone, as well as the M168 side chain from the adjacent β4-strand. This results in a shift in the backbone of the β3 and β4 strands, pushing the side chains of the residues lining the active site facing (Y157, Y159, I161, Y162 and N169) deeper into the active site. This creates a narrower and less deepened active site cleft, as shown in Figure 3C and Figure 4C.

In both WT and mutant species, the C-terminal region or the protein is highly mobile during all simulations, easily dissociating from the larger protein globule. As expected, in the E239K apoenzyme simulations, the substitution of a side chain that forms in a salt bridge leads to a decrease in the number of heavy atom contacts between the α10-helix and the rest of the strongly positively charged AAG globule surface (Figure 3D and Figure 4D) [46]. In contrast to the other two variants, the active site of the apoenzyme remains relatively unaffected.

### 2.2. Dissociation Constant of the Enzyme–Substrate Complex

To test the effect of selected AAG polymorphic variants on the enzyme–DNA binding step, the enzyme–substrate complexes were separated using a non-denaturing polyacrylamide gel. Selected mutant variants contain substitutions of amino acid residues that are not directly involved in DNA coordination. However, a decrease in thermal stability could affect the formation of the enzyme–substrate complex. Two structurally different DNA lesions, N^6^–ethenoadenosine (εA) and hypoxanthine (Hx), were chosen as substrates. Both damaged DNA bases are processed by the WT AAG enzyme.

**Figure 5:**
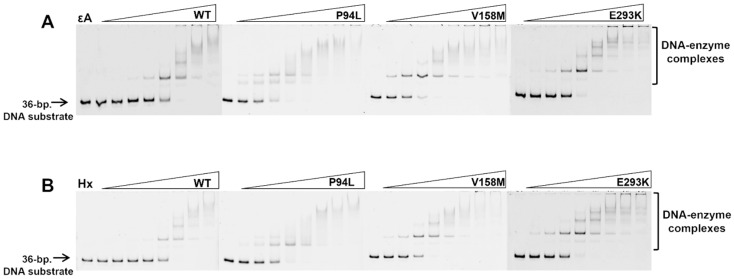
Binding of WT AAG and polymorphic variants to a DNA substrate containing εA (**A**) or Hx (**B**) residue. Experiments were performed using serial dilutions of the enzymes: WT AAG from 38.7 nM to 4.95 μM, P94L from 0.12 μM to 15.6 μM, V158M from 0.3 μM to 38.3 μM, E293K from 0.1 μM to 13.9 μM. The concentration of the DNA substrate was 50 nM.

**Figure 6:**
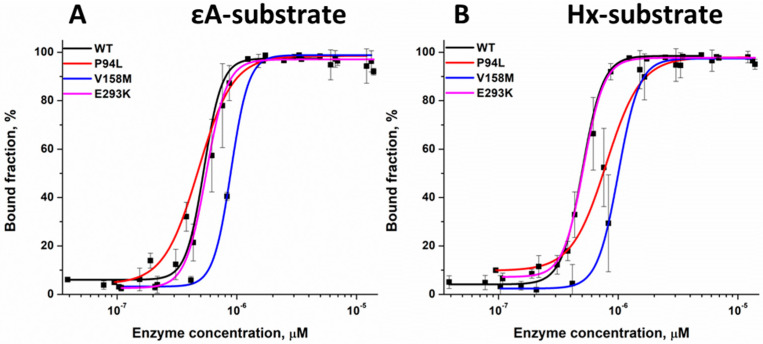
Efficiency of enzyme–substrate complex formation by polymorphic variants and WT AAG with a DNA substrate containing εA (**A**) and Hx (**B**) residue. The concentration of WT AAG was varied from 38.7 nM to 4.95 μM, P94L from 95 nM to 12 μM, V158M from 0.1 μM to 13.3 μM, E293K from 0.1 μM to 13.9 μM. The concentration of DNA substrate was 50 nM.

Formation of an enzyme–DNA complex was detected for all polymorphic variants and WT AAG using both DNA substrates. Notably, both the WT enzyme and the polymorphic variants formed several complexes with varying electrophoretic mobilities (Figure 5). In the case of the E293K mutant variant, up to six bands were observed. Each dissociation constant determination experiment was repeated at least three times; the gel processing results are shown in Figure 6. The resulting dependencies were approximated using Equation (2) to obtain dissociation constant values (Table 2).

Despite the differences in melting temperatures, the dissociation constant values for the studied polymorphic variants were close to those of the WT enzyme. An exception was found for the V158M variant, for which the dissociation constant values for complexes with DNA containing εA and Hx were two times higher than those for the WT enzyme.

### 2.3. Comparison of Glycosylase Activity

**Figure 7:**
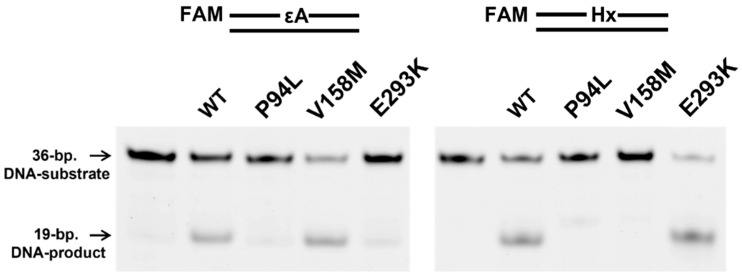
Comparative analysis of the glycosylase activity of WT AAG and polymorphic variants P94L, V158M, and E293K. The enzyme concentration in the reaction mixture was 2 μM, and the DNA substrate concentration was 0.25 μM. The reaction was conducted for 30 min at 37 °C.

**Figure 8:**
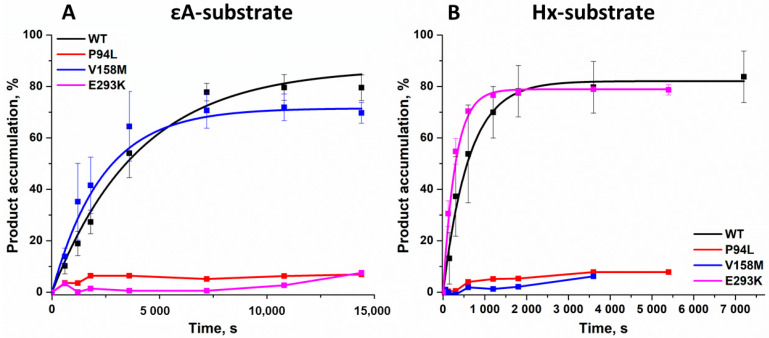
Time dependence of reaction product accumulation for polymorphic variants P94L, V158M, E293K, and WT AAG. (**A**) Reaction product accumulation for εA substrate and (**B**) reaction product accumulation for Hx substrate. The enzyme concentration in the reaction mixture was 2 μM; the DNA substrate concentration was 0.25 μM.

To compare the effects of amino acid substitutions on the enzyme’s ability to remove damaged bases from DNA, a glycosylase reaction was performed. The reaction products were then further treated with a NaOH solution to create a break in the DNA strand. The reaction mixture was separated on a denaturing polyacrylamide gel (Figure 7). The resulting gel images were processed using the GelPro software, v. 4. The time dependence of reaction product formation was approximated using Equation (3) (Figure 8) to obtain the observed rate constant *k*_obs_ (Table 3).

Despite the lack of differences in DNA substrate binding efficiency, the AAG polymorphic variants showed activities that differed from those of WT. It was found that the P94L mutant was unable to remove either εA or Hx residues from DNA. In both cases, low activity was recorded—less than 10% of the DNA substrate was cleaved over a period of more than one hour. This suggests that replacing Pro-94 with Leu inactivates the enzyme.

The substitution of Val-158 with Met had no effect on the removal of the εA residue from DNA. However, this polymorphic variant was inactive against the Hx substrate.

Unlike the V158M variant, the E293K variant was active against DNA containing the Hx residue but was inactive against the εA substrate.

**Figure 9:**
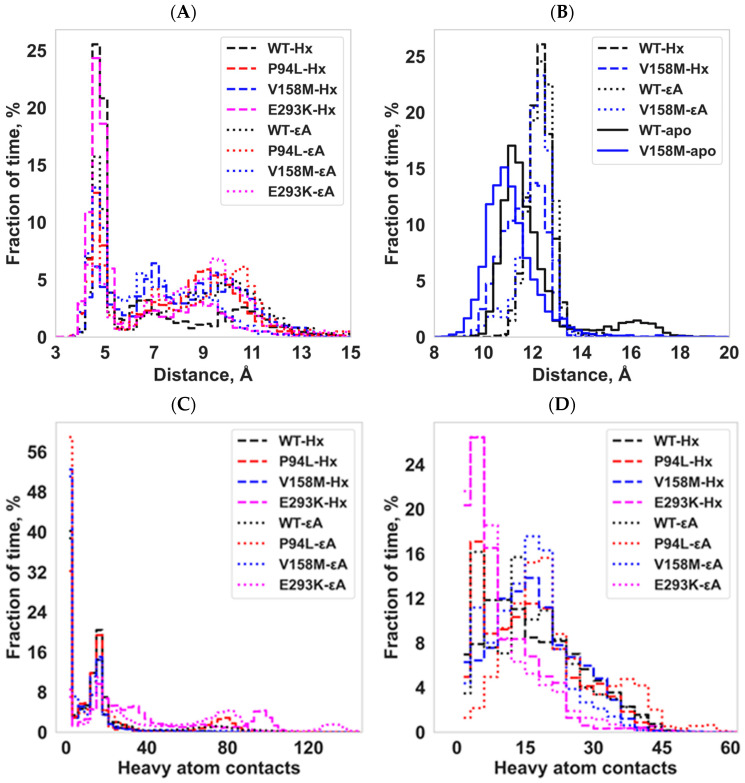
Distance between the R138 CZ atom and the phosphate backbone P atom upstream of the lesion site (**A**), distance between the Cα atoms of V/M158 and H136 (**B**), heavy atom contacts between the C-terminal region (aa 289–298) and DNA (**C**), heavy atom contacts between the C-terminal region (aa 289–298) and the rest of the protein globule (aa 82–260) (**D**).

A notable difference in protein–DNA contacts was observed during the simulations of the WT lesion recognition complex with εA and Hx containing DNA. In the Hx-DNA complex, the R138 side chain preferentially interacts with the lesion-containing strand, forming a salt bridge with the phosphate group of the upstream nucleotide, whereas in the εA-DNA complex, it either shifts to a phosphate group of the complementary stand or positions itself between the two strands, as observed in the crystal structures. Among the mutant species, this effect persists only in the E293K Hx-DNA complex, but not in the εA-DNA one (Figure 9A).

The bulkier εA appears to stabilize the active site of the V158M, with the distance between the backbone carbonyl atoms of V/M158 and H136, another metric for active site volume, maintained at levels similar to those in the WT protein (Figure 9B).

Abolition of the E293-R280 interaction results in a more positively charged C-terminal region that dissociates from the protein and engages the DNA more prominently. While in the WT and the other two variants, only the R290 side chain is capable of maintaining salt bridge contacts with the DNA backbone, the addition of K293 drastically alters the local structure (Figure 9C,D).

## 3. Materials and Methods

### 3.1. Site-Directed Mutagenesis and DNA Substrates

To create vectors containing single nucleotide mutations, the pET-11a plasmid containing the Δ80 AAG gene was used. Substitutions P94L, V158M and E293K within the AAG coding sequence were generated by site-directed mutagenesis. A polymerase chain reaction was performed according to the standard protocol for Phusion™ High-Fidelity DNA polymerase (ThermoFisher, Waltham, MA, USA). Primer sequences are presented in Table 4. The gene sequence was confirmed by DNA sequencing at the SB RAS Genomics Core Facility (ICBFM SB RAS, Novosibirsk, Russia).

The 2′-deoxyoligonucleotides used in this study are listed in Table 5. DNA oligonucleotides were obtained from Biosset Ltd. (Novosibirsk, Russia), the 5′-end FAM-labeled 2′-deoxyoligonucleotides were purified by 20% denaturing PAGE. DNA substrates for dissociation constant measurements and the glycosylase assay were prepared by mixing equimolar amounts of complementary strands, followed by annealing at 95 °C. The damaged strand contained either 1, N^6^–ethenoadenosine (εA) or hypoxanthine (Hx) residues.

### 3.2. Protein Purification

The enzyme induction was carried out in *E. coli* Rosetta 2 (DE3) cells transformed with the pET11a plasmid, carrying either the WT enzyme gene or a gene with a single nucleotide mutation. The 2 L of LB medium containing 100 mg/mL Amp were inoculated with the corresponding night culture. The cells were grown at 37 °C and 220 rpm to an OD of 0.6–0.8 o.u./mL. The incubator temperature was then reduced to 16 °C, and an IPTG solution was added to a concentration of 0.2 mM. The cells were grown at 16 °C and 220 rpm for 20 h.

The cells were pelleted by centrifugation for 10 min at 5000 *g*, after which the culture fluid was decanted. A cell suspension was resuspended in 20 mL of buffer A (20 mM HEPES-KOH (pH 7.8), 40 mM NaCl) and a protease inhibitor cocktail (Inhibitor cocktail, Complete, Mannheim, Germany). Resuspended cells were lysed using a French press. The lysate was centrifuged at 4 °C and 40,000 rpm for 40 min. The supernatant was decanted into a prepared clean container and placed on ice. Chromatography was performed on a Q-Sepharose column. The lysate was adjusted to a 100 mM NaCl, then loaded onto the column and washed with buffer B (20 mM HEPES-KOH (pH 7.8), 100 mM NaCl) at a flow rate of 2 mL/min. The collected fractions containing AAG enzyme were loaded on SP Hi-trap column equilibrated with a buffer containing 20 mM HEPES, 100 mM NaCl, pH = 7.8. An AKTA START system was utilized for chromatography, employing a buffer gradient that transitioned from 100 mM NaCl to 500 mM NaCl. AAG-containing fractions were collected and subsequently purified through a BIOFIL 50,000 MWCO filter (Guangzhou, China). To increase the concentration of AAG, the collected fraction was processed using Amicon 3K filters (Burlington, MA, USA) and then diluted to achieve a final glycerol concentration of 50%. Gel electrophoresis was employed to assess the purity of the isolated enzymes, while the enzyme concentration was quantified using the Bradford assay. Purified enzyme fractions were stored at −20 °C.

### 3.3. Thermal Stability Measurement

Melting curves of enzyme stocks were obtained using a Quant Studio 5 DNA amplifier (Applied Biosystems, Waltham, MA, USA) and the fluorescent probe ProteOrange (Lumiprobe, Moscow, Russia). The 50 μL probe of AAG enzymes were diluted in a thermal melt buffer (50 mM Tris-HCl, 50 mM KCl, 10 mM EDTA, pH 7.5, 5× ProteOrange) to the final concentration of 5 μM. Each melting curve was approximated using the Boltzmann equation (Equation (1)) to calculate the enzyme’s melting point:

F = Fu + (Fb − Fu)/{1 + exp(Tm − x/slope)},(1)

where F is the fluorescence intensity of the ProteOrange, x is the temperature, Fu is the fluorescence baseline at a low temperature, Fb is the fluorescence maximum at a high temperature, and Tm is the melting temperature of the protein.

### 3.4. DNA Binding Assay

The dissociation constant *K*_d_ of the AAG enzyme complex with DNA was determined using an electrophoretic mobility shift assay (EMSA) in non-denaturing polyacrylamide gel. The reaction was conducted in a buffer solution containing 50 mM Tris-HCl (pH 7.5), 50 mM KCl, 1 mM Na_2_EDTA, 5 mM MgCl_2_, 1 mM DTT, and 7% glycerol. A series of enzyme concentrations was achieved through serial two-fold dilutions. The analyzed mixture was prepared by adding 5 μL of the 100 nM DNA substrate to 5 μL of the diluted enzyme solution to a final DNA concentration of 50 nM. The samples were incubated for 15 min on ice and applied to 10% non-denaturing PAGE (the ratio of acrylamide to N, N’-methylenebisacrylamide was 75:1). To determine the dissociation constant *K*_d_, the resulting gel was visualized in the VersaDoc gel documentation system (Bio-Rad Laboratories, Hercules, CA, USA). The results were processed using Gel-Pro Analyzer 4 (Media Cybernetics, Rockville, MD, USA). The dissociation constant *K*_d_ for each enzyme–DNA complex was calculated using OriginPro 8 using Equation (2):

Enzyme–substrate complex (%) = Fu + (Fb − Fu)/(1 + (*K*_d_/[AAG])^h^) (2)

where h is the Hill coefficient, Fu is the correction for background illumination, and Fb is the maximum intensity of the complex.

### 3.5. DNA Glycosylase Assays

WT AAG or mutant variants were mixed with DNA substrates to the final concentrations of 2 μM and 0.25 μM, respectively. The reaction mixture was incubated at 37 °C in 50 mM Tris-HCl (pH 7.5), 50 mM KCl, 1 mM Na_2_EDTA, 5 mM MgCl_2_, 1 mM DTT, and 7% glycerol buffer. DNA was chemically cleaved at abasic sites with 1 M NaOH at 72 °C for 15 min and neutralized with 1 M HCl. Reactions were analyzed by 20% denaturing PAGE.

The gels obtained were examined utilizing the VersaDoc gel documentation system from Bio-Rad Laboratories (Hercules, CA, USA). The data was analyzed with Gel-Pro Analyzer 4 (Media Cybernetics, USA), and the extent of substrate conversion was calculated by taking the ratio of the product peak area to the total area of both the product and the initial substrate peaks.

To quantitatively assess the catalytic activity of various polymorphic variants, the rate of product accumulation over time was examined. The final determination of the observed reaction rate constant was carried out using OriginPro 8 software, where product accumulation was plotted against reaction time. The resulting data were fitted using Equation (3):

[Product] = [S] × (1 − exp(−*k*_obs_ × t)) (3)

where [S] is the initial concentration of the substrate, t is the reaction time, and *k*_obs_ is the observed rate constant of the chemical reaction.

### 3.6. Molecular Dynamics Simulations

AAGΔ79 lesion recognition complex (LRC) structures were built based on the crystal structure of hAAG in complex with εA-containing DNA [38]. Unresolved C-terminal amino acid residues were adapted from an AlphaFold2 structure [47,48], and unresolved flexible loops were rebuilt with Modeller [49]. The apoenzyme AAG model was based on an AlphaFold2 structure. Histidine side chain protonation states were assigned using H++ server [50]. Simulation setup and simulations were performed in the GROMACS 2025 MD package [51]. The initial structures were parametrized using an AMBER 14SB-OL24 force field for the protein and the DNA primer [52,53,54], solvated in a dodecahedral cell filled with TIP3P model water [55] and neutralized with 100 mM of K+ and Cl− ions with Joung–Cheatham parameters [56]. The parameters for εA and Hx bases were adapted from [57,58]. The non-bonded interactions cutoff was set at 1.0 nm, and long-range electrostatic interactions were treated using a smooth particle mesh Ewald method. Hydrogen atom covalent bonds were treated using LINCS solver [59], enabling a 2 ns time step. Distance restraints were applied to the terminal base pairs of the 18 bp truncated substrate DNA. The steepest descent energy minimization was followed by 1 ns NVT equilibration with V-rescale thermostat and 1 ns NPT equilibration with C-rescale barostat at 310 K system temperature [60,61]. Post-equilibration unrestrained MD simulations were run five times for 300 ns each. Trajectories were processed using integral GROMACS 2025.4 tools and MDtraj v. 1.11 [62]. Representative snapshots were obtained by clustering aggregate trajectories while excluding highly flexible regions and the first 25 ns of each trajectory. The cavity volume was estimated using MDpocket [63].

## 4. Conclusions

In this study, three rare single nucleotide polymorphic variants of the human AAG enzyme (P94L, V158M, and E293K) were comprehensively investigated. Previously, we identified these SNPs as having a high predicted deleterious potential. Using a combination of in vitro methods (measuring thermal stability, DNA binding, and enzymatic activity) and molecular dynamics modeling, we found that even amino acid substitutions located far from the active site can dramatically alter the functional properties of the enzyme.

All three mutations reduce the protein’s melting temperature by 7–10 °C compared to the WT, indicating a destabilizing effect on the globular structure of AAG. Despite these structural alterations, the P94L, V158M, and E293K variants retain their ability to bind to damaged DNA containing either εA or Hx residues, with dissociation constants *K*_d_ close to those of the WT AAG. The most notable finding is the loss or switch of substrate specificity: P94L is completely inactive for both tested substrates (εA and Hx); V158M remains active against εA but unable to remove the Hx; and E293K, in contrast, is active against the Hx but inactive against the εA. These data demonstrate that point substitutions can not only reduce the overall activity but also qualitatively alter the enzyme’s substrate specificity profile, an outcome that cannot be predicted by in silico methods alone. It is worth noting that all biochemical characteristics were obtained in a cell-free system using recombinant proteins, and our data do not reflect the influence of the cellular microenvironment. Thus, the observed decrease in activity in vitro may not fully correlate with the physiological phenotype in vivo. Nevertheless, our biochemical study demonstrates that even single rare substitutions can significantly suppress enzyme activity. This is critically important because it indicates the vulnerability of this metabolic pathway: regardless of the frequency of the allele in the population, if a mutation occurs in a particular patient, it objectively disrupts the function of the protein. Thus, we provide an experimental foundation for understanding pathogenesis rather than a statistical tool for population prediction.

Variants P94L, V158M, and E293K have been found to be common in various types tumors, including endometrioid carcinoma, renal cell carcinoma, and cervical cancer. Characterization of their functional impairments allows these SNPs to be considered as potential biomarkers for assessing the risk of developing certain types of cancer, as well as for predicting tumor responses to DNA-damaging therapies (e.g., alkylating agents). The experimental approach proposed in this study (a combination of measuring thermal stability, binding, and activity of variants on two types of lesions) can be adapted to screen for functional variants in any new clinically significant AAG variants, as well as to categorize unclassified SNPs from population databases. The fact that some variants (e.g., V158M and E293K) retain partial activity that varies across different lesions is important for the development of AAG inhibitors. Understanding the structural differences between these variants will enable the design of selective molecules that will inhibit pathologically overexpressed AAG in tumors without affecting normal variants. This study reveals the molecular mechanisms of dysfunction of clinically significant AAG variants.

## Figures and Tables

**Table 1:** Calculated melting point values of WT AAG and mutant variants.

Mutant Form	Tm, °C
WT	42 ± 1
P94L	35 ± 1
V158M	32 ± 1
E293K	33 ± 1

**Table 2:** Calculated dissociation constants of the enzyme–DNA complex.

Mutant Form	εA-Substrate *K*d, µM	Hx-Substrate *K*d, µM
WT	0.53 ± 0.08	0.50 ± 0.08
P94L	0.48 ± 0.03	0.8 ± 0.1
V158M	0.9 ± 0.1	1.0 ± 0.2
E293K	0.6 ± 0.1	0.51 ± 0.03

**Table 3:** Calculated values of the observed reaction rate constant *k*_obs_, s^−1^.

Mutant Form	εA Substrate *k*_obs_, s^−1^	Hx Substrate *k*_obs_, s^−1^
WT	(2.5 ± 0.4) × 10^−4^	(16 ± 2) × 10^−4^
P94L	n/a	n/a
V158M	(4.3 ± 0.4) × 10^−4^	n/a
E293K	n/a	(34 ± 2) × 10^−4^

**Table 4:** Primers for site directed mutagenesis. F primer—forward, R—reverse.

Mutation	Codon Substitution	Primer Sequences
P94L	CCG→CTG	F: GGTTGGAGTTTTTCGACCAGCTGGCAGTCCCCCTGGCCC R: GGGCCAGGGGGACTGCCAGCTGGTCGAAAAACTCCAACC
V158M	GTG→ATG	F: GAAGCCGGGGACCCTGTACATGTACATCATTTACGGCATG R: CATGCCGTAAATGATGTACATGTACAGGGTCCCCGGCTTC
E293K	GAG→AAG	F: GGTCGACAGAGTGGCTAAGCAGGACACACAGGCC R: GGCCTGTGTGTCCTGCTTAGCCACTCTGTCGACC

**Table 5:** The sequence of the 2′-oligodeoxyribonucleotides employed for glycosylase activity and DNA binding assays.

Name	Sequence
Damaged strand	FAM 5′-GCCTCGCAGCCGTCCAACC**N**TAGTCACCTCAATCCA-3′
Complimentary strand	5′-TGGATTGAGGTGACTATGGTTGGACGGCTGCGAGGC-3′

**N = εA/Hx.**

## Data Availability

Raw experimental data are available from O.A.K. and A.A.K. upon request.
